# Sugarcane calcineurin B-like (*CBL*) genes play important but versatile roles in regulation of responses to biotic and abiotic stresses

**DOI:** 10.1038/s41598-019-57058-7

**Published:** 2020-01-13

**Authors:** Weihua Su, Long Huang, Hui Ling, Huaying Mao, Ning Huang, Yachun Su, Yongjuan Ren, Dongjiao Wang, Liping Xu, Khushi Muhammad, Youxiong Que

**Affiliations:** 1 Key Laboratory of Sugarcane Biology and Genetic Breeding, Ministry of Agriculture/National Engineering Research Center for Sugarcane, Ministry of Science and Technology, Fujian Agriculture and Forestry University, Fuzhou, 350002 China; 2 Department of Genetics, Hazara University, Mansehra, Pakistan; 3 Guangxi Collaborative Innovation Center of Sugarcane Industry, Guangxi University, Nanning, 530005 China

**Keywords:** Abiotic, Biotic

## Abstract

Free calcium ions are common second messengers in plant cells. The calcineurin B-like protein (CBL) is a special calcium sensor that plays an important role in plant growth and stress response. In this study, we obtained three *CBL* genes (GenBank accession nos. KX013374, KX013375, and KX013376) from sugarcane variety ROC22. The open reading frames of *ScCBL* genes ranged from 642 to 678 base pairs in length and encoded polypeptides from 213 to 225 amino acids in length. ScCBL2-1, ScCBL3-1, and ScCBL4 were all located in the plasma membrane and cytoplasm. *ScCBL*2-*1* and *ScCBL*3-*1* expression was up-regulated by treatment with salicylic acid (SA), methyl jasmonate (MeJA), hydrogen peroxide (H_2_O_2_), polyethylene glycol (PEG), sodium chloride (NaCl), or copper chloride (CuCl_2_). *ScCBL*4 expression was down-regulated in response to all of these stresses (abscisic acid (ABA), SA, MeJA, and NaCl) except for H_2_O_2_, calcium chloride (CaCl_2_), PEG, and CuCl_2_. Expression in *Escherichia coli* BL21 cells showed that ScCBLs can enhance tolerance to NaCl or copper stress. Overexpression of *ScCBLs* in *Nicotiana benthamiana* leaves promoted their resistance to infection with the tobacco pathogen *Ralstonia solanacearum*. The results from the present study facilitate further research regarding *ScCBL* genes, and in particular, their roles in the response to various stresses in sugarcane.

## Introduction

Calcium (Ca^2+^) is an important inorganic nutritive element and a ubiquitous second messenger^1^. Ca^2+^ not only plays a vital role in maintaining the stability of the cell wall, cell membrane, and membrane binding proteins, but also is widely involved in the regulation and control of plant growth and development, as well as response to external environmental stimuli^2^. In plants, intracellular Ca^2+^ sensors accurately recognize specific Ca^2+^ signatures that are generated in response to different external stimuli^3^. Under adverse conditions, cell signal transduction receptors on the plant cell membrane recognize the stimulus signal, and then activate the Ca^2+^ channel protein through phosphorylation, thereby leading to an instantaneous increased in the Ca^2+^ concentration in the cytoplasm, which produces the “Ca^2+^ signal”^4^. Ca^2+^ sensors in plants detect this stress-induced Ca^2+^ signal and deliver it to downstream effectors, activating a signal cascade reaction that regulates resistance and tolerance^5^. Ca^2+^ sensors can be divided into two main types according to their structural features. One type is the sensor responders, including calcium-dependent protein kinases and calmodulins, which have all the functions of Ca^2+^ sensor relay proteins, as well as the kinase activity^6^. The other type is the sensor relays, including calmodulin-like proteins and CBL, which do not have kinase activity. Sensor relays can specifically target downstream proteins to transfer the perceived Ca^2+^ signals in response to various environmental stimuli^6^.

CBL, a Ca^2+^ sensor relay protein that is expressed in a wide range of plants, can interact with a family of serine-threonine protein kinases known as CBL-interacting protein kinases (CIPKs)^7^. CBLs were initially identified in *Arabidopsis thaliana* and are closely related to both the regulatory B subunit of calcineurin and neuronal calcium sensors in animals^8^. *CBL*s have been found in many terrestrial plants, such as mosses, ferns, gymnosperms, monocots, and dicots^9^. *CBL*s are a multigene family. The *A. thaliana*, *Oryza sativa*, and *Populus trichocarpa* genomes are each predicted to contain approximately 10 *CBL* family members^10–12^. Eight *CBL* genes have been identified in *Sorghum biocolor*^13,14^. In addition, *CBL* genes have been investigated in *Brassica napus*^15^, *Solanum melongena*^16^, and other plant species^17,18^. CBL proteins contain a classical EF-hand helix-loop-helix motif with a 12-residue loop^19^. In EF-hand motifs, the Ca^2+^-binding sites are located at residues 1 (X), 3 (Y), 5 (Z), 7 (Y), 9 (X), and 12 (Z)^10,19^. Different CBL proteins have different degrees of variation in the EF-hand structure, but the number of EF-type regions and the distance between them is the same in all known CBL proteins^11^.

The function of *CBL* genes has been studied in *A. thaliana*, *O. sativa*, and other plant species. In *A. thaliana*, *AtCBLs* play a role in the response to multiple abiotic stresses^20–22^. For instance, *AtCBL1* functions as a positive regulator in response to salt and drought but as a negative regulator in response to cold^20^. Abscisic acid (ABA) is a signaling molecule that plays a role in the plant response to aging and stress^23^. *AtCBL9* is a common element in the ABA signaling and stress-induced ABA biosynthesis pathways^21^. Ten *OsCBL* genes in rice are expressed in various organs at the adult stage and have also been found to respond to different stress conditions [sodium chloride (NaCl), polyethylene glycol (PEG), and cold]^24^. In addition, *OsCBL8* overexpressing transgenic rice seedlings showed more tolerance to salt stress than non-transgenic seedlings^24^. *S. bicolor CBL* genes are thought to regulate sodium carbonate stress-specific cellular adaptation responses and influence the plant growth and developmental patterns^14^. Analysis of *CBL* transcripts in *Populus euphratica* under abiotic stress suggested that seven *CBL* (*PeCBL1*, *2*, *3*, *4*, 5, 9, and 10) members may play important roles in responding to specific external stimuli^12^.

Sugarcane (*Saccharum* spp.) is an economically attractive polyploid C4 grass that is used not only to produce approximately 60% of the world’s sugar but also to produce ethanol, a low-carbon-emission fuel^25^. To date, there have been few reports on *CBL* genes in sugarcane^26–28^. Zhang (2013) cloned five *CBL* genes (GenBank accession Nos. KC800815, KC800816, KC800817, KC800818, and KC800819) from *Saccharum* hybrid variety GT28 and found that *CBL5* and *CBL6* may play key roles in adaptation to low temperatures^28^. Using real-time quantitative polymerase chain reaction (qRT-PCR) analyses, Ling *et al*. (2018) found that *SsCBL1* and *SsCBL6* play important regulatory roles in response to a variety of stresses (low potassium, drought, and salt)^26^. Yeast two-hybrid assays showed that ScCIPK8 interacts with ScCBL1, ScCBL3, and ScCBL6^27^. In the past 15 years, ROC22 is the most widely cultivated sugarcane in China due to its high yield and high sugar and good ratoon properties. Previous research found that ROC22 can well resist infection by Pokkah boeng disease^29,30^. Lan *et al*. (2014) found that ROC22 has better drought tolerance compared to five sugarcane varieties^31^. However, a systematic analysis of *CBL* genes in sugarcane variety ROC22 especially on the view of function differentiation, however, has not yet been reported.

In this study, we successfully cloned three sugarcane *ScCBL* genes by reverse transcription-PCR (RT-PCR) and subjected the cloned sequences to bioinformatics analysis. The expression patterns of these three *ScCBL* genes in different sugarcane tissues and under various exogenous stresses were investigated by qRT-PCR. In addition, we assessed the subcellular localization of these ScCBL proteins and analyzed their function by expression in *Escherichia coli* BL21 and transient expression in *N. benthamiana*. This study aims to provide useful information about the sequence characteristics of these three ScCBL proteins as well as their expression patterns in response to phytohormones and various stresses. This increased knowledge of *ScCBL* genes could be applied by sugarcane breeder to develop resistant variety.

## Materials and Methods

### Plant materials and treatments

The sugarcane variety ROC22 were provided by the Key Laboratory of Sugarcane Biology and Genetic Breeding, Ministry of Agriculture (Fuzhou, China).

We sampled sugarcane tissues as described by Wang^32^. Selecting three healthy and mature ROC22 sugarcane stalks with uniform growth rates from the field. The bud, stem pith, stem skin, meristem, and the youngest fully expanded viz + 1 leaf with a visible dewlap (the collar between the leaf blade and sheath) were sampled. These tissues were wrapped, frozen in liquid nitrogen, and stored at −80 °C until total RNA extraction. We performed the sampling after abiotic stress treatment as follows^33^: uniform four-month-old cultured ROC22 plantlets were transferred to water for one week and then treated with eight exogenous treatments, including 100 μM ABA, 5 mM salicylic acid (SA), 100 μM methyl jasmonate (MeJA), 50 μM calcium chloride (CaCl_2_), 10 μM hydrogen peroxide (H_2_O_2_), 25% PEG 8000, 250 mM NaCl, or 100 mM copper chloride (CuCl_2_), by root dipping at 28 °C with 16 h light and 8 h darkness^34^. Whole plantlets treated with CaCl_2_, SA, MeJA, or ABA were collected at 0 h, 3 h, 6 h, and 12 h. Plantlets treated with H_2_O_2_, PEG, or NaCl were collected at 0 h, 6 h, 12 h, and 24 h. Plantlets subjected to Cu stress were harvested at 0 h, 12 h, 24 h, and 48 h. Each treatment group contained three biological replicates, and plantlets were stored at −80 °C until extraction of total RNA.

### Total RNA extraction and the first-strand cDNA synthesis

Total RNA were extracted from all samples with Trizol^®^ Reagent (Invitrogen, Carlsbad, CA, USA) according to the manufacturer’s specifications. RNA samples with an OD_260_/OD_280_ of between 1.8 and 2.0 were selected and treated with DNase I (Promega, Madison, WI, USA) to remove DNA contamination. First-strand cDNA was synthesized using a Prime-Script^TM^ RT Reagent Kit (TaKaRa, Dalian, China) according to the manufacturer’s protocol, and then checked by 1% agarose gel electrophoresis.

### Isolation of sugarcane *ScCBL* genes and gateway entry vector construction

The *Z. mays* sequence (GenBank Acc No. NM_001155706) which derived from ZmCBL3 (GenBank Acc No.EU962348.1) was used as a probe and the NCBI BlastN tool was applied to retrieve homologous EST sequences in the sugarcane genome. The BioEdit Contig Assembly Program (CAP) was employed to assemble one sugarcane CBL sequence (*ScCBL2-1*). The other two sugarcane CBL sequences (*ScCBL3-1* and *ScCBL4*) were selected from our previous transcriptome data of sugarcane infected with *Sugarcane Mosaic virus*^35^. The specific primers were designed using Primer 5.0 and the NCBI primer designing tool (http://www.ncbi.nlm.nih.gov/tools/primer-blast/) (Table S1).

The RT-PCR reactions (Table S2) were designed according to the specifications for *Ex* Taq (TaKaRa, Dalian, China). Subsequently, the PCR products were detected by 1% agarose gel electrophoresis and purified, ligated into the pMD-19-T vector, transformed into *E. coli* DH5α and then sequenced (Shenggong, Shanghai, China).

The *ScCBL* ORFs were amplified from pMD19-T-*ScCBLs* with Gateway entry adapters attB1 and attB2 using the primers shown in Table S1. The PCR conditions are shown in Table S2. The PCR amplification products were gel**-**purified and transformed into the Gateway^@^ donor vector pDONR221 (Invitrogen) following the manufacturer’s instructions and using Gateway^®^ BP Clonase^TM^ II Enzyme Mix (Invitrogen). The BP reaction mixtures were transformed into competent *E. coli* DH5α cells and sequenced (Sangon, Shanghai, China). Then the verified pDONR221-*ScCBL* plasmids were used to construct the expression vectors.

### Sequence analysis of ScCBL genes

The ORF Finder (http://www.ncbi.nlm.nih.gov/gorf/gorf.html) was used to translate and analyze the three *ScCBL* genes. Molecular weight (MW), isoelectric points (pIs), and grand average of hydropathicity (GRAVY) of each protein sequence were analyzed by ExPASY (http://web.expasy.org/protparam/). The DNAMAN software was applied to conduct multiple alignment. The phylogenetic tree was constructed by the neighbor-joining bootstrap method (1,000 replicates) using the MEGA X program^36^.

### qRT-PCR analysis

The 7500 qRT-PCR system (Applied Biosystems, San Francisco, CA, USA) was applied to detect and analyze the expression of *ScCBL* genes in different sugarcane tissues and under various exogenous stresses. The qRT-PCR primers (Table S1) were designed using Beacon Designer 8.12 software. Cullin (*CUL*)^37^ and clathrin adaptor complex (*CAC*)^37^ were employed as the internal controls (Table S1). The 20 μL qRT-PCR reaction contained 10 μL SYBR Green Master Mix, 0.8 μL each of the 10 μM primers, 1.0 μL cDNA templates (20 × diluted cDNA), and 7.4 μL double distilled water. Each qRT-PCR reaction was repeated three times, and the conditions were as follows: 50 °C for 2 min, 95 °C for 10 min, 40 cycles of 95 °C for 15 s, and 60 °C for 1 min. The 2^-ΔΔCt^ method was used to analyze the qRT-PCR data^38^ and the statistical analysis was conducted by using Data Processing System v9.50 software (China). Data were expressed as the mean ± standard error (SE). Significance (p < 0.05) was calculated using one-way analysis of variance (ANOVA), followed by Duncan’s new multiple range test.

### Subcellular localization assay

The pFAST-R05-*ScCBL2–1*-GFP, pFAST-R05-*ScCBL3-1*-GFP, and pFAST-R05-*ScCBL4*-GFP vectors were constructed by LR reaction using the LR Clonase^TM^ II Enzyme Mix (Invitrogen) according to the manufacturer’s instructions. The three recombinant *ScCBL*-GFP vectors were transformed into competent *Agrobacterium tumefaciens* GV3101 cells. The *Agrobacterium*-mediated transient expression in *N. benthamiana* leaves was performed according to the method described by Su *et al*.^39^. After infiltration for 48 h, the subcellular localization of the fusion protein were visualized by laser scanning confocal microscopy (Leica TCS SP5, Wetzlar, Germany).

### Expression in *E. coli* BL21 (DE3) cells

Prokaryotic expression vectors were constructed based on pEZYHb from the pDONR221-*ScCBL* plasmids by LR reaction. The recombinant pEZYHb-*ScCBL* plasmids and the empty vector pEZYHb (control) were transformed into competent *E. coli* BL21 (DE3) cells for the prokaryotic expression experiments.

A spot assay was conducted to characterize the expression of *ScCBLs* in competent *E. coli* BL21 cells in response to NaCl and CuCl_2_ stresses. When the OD_600_ of *E. coli* BL21 cells containing pEZYHb-*ScCBLs* or pEZYHb (control) in LB medium (containing 80 μg/mL ampicillin) reached 0.6, 1.0 mM Isopropyl β-D-thiogalactoside (IPTG) was added, and the cells were cultured at 37 °C for another 12 h. The concentration of the cultures was adjusted to OD_600_ = 0.6, and the samples were then diluted 10^−3^ and 10^−4^ in LB medium^40^. Next, 10 μL from each of the 10^−3^ and 10^−4^ dilutions was spotted on LB agar plates. For the salt tolerance assay, we prepared LB media with 250 mM, 500 mM, or 750 mM of NaCl. For the heavy metal tolerance assay, we added 250 μM, 500 μM, or 750 μM of CuCl_2_ to the LB media^36^. All of the plates were incubated at 37 °C overnight and then photographed.

### Transient assay for *ScCBL* genes in *N. benthamiana* leaves

To understand how *ScCBL* expression changes in response to pathogen infection and whether the plant hypersensitive reaction is also activated, pEarleyGate 203-*ScCBLs* overexpressing vectors were constructed using the Gateway cloning technique. Competent *Agrobacterium* GV3101 cells were transformed with recombinant pEarleyGate 203-*ScCBL* plasmids. The empty pEarleyGate 203 vector was transformed into *Agrobacterium* GV3101 cells for use as a control. The cells were then cultured in LB liquid medium (supplemented with 50 μg/mL kanamycin and 35 μg/mL rifampicin) overnight at 28 °C. After incubation, the cells were centrifuged and resuspended in MS liquid medium (containing 200 μM acetosyringone) at an OD_600_ of 0.8. After infiltration into the *N. benthamiana* leaves, the plants were cultured at 24 °C for 24 h (16 h light/8 h darkness)^39^. RT-PCR was exploited to detect whether *ScCBL* genes have been overexpressed in *N. benthamiana*, with the RNA of treated leaves and *ScCBL* genes specific primers (*gScCBL2-1*, *gScCBL3-1*, and *gScCBL4*, Table S1), and the *NtEF1-α* was treated as control. The treated *N. benthamiana* leaves were used for the transcriptional analysis of the eight tobacco immunity-associated marker genes (Table S1)^41^.

To analyze the inhibitory effect of *ScCBL* genes on pathogen infection, *Ralstonia solanacearum* was cultured to an OD_600_ of 0.8 in potato dextrose water (PDW) liquid medium at 28 °C. Then, *N. benthamiana* leaves that had been infiltrated with pEarleyGate 203-*ScCBLs* or pEarleyGate 203 for 24 h were infected with *R. solanacearum*. All of the treated plant materials were cultured at 24 °C (16 h light/8 h darkness) for 7 days and then photographed. DAB and trypan blue staining were utilized to analyze the *Agrobacterium***-**infiltrated leaves as described by Liu *et al*.^33^.

## Results

### Identification of *CBL* genes in sugarcane

Three *ScCBL* genes were successfully amplified by RT-PCR from sugarcane variety ROC22. According to the homology with AtCBLs (Figure S1), three *ScCBL* genes were designated as *ScCBL2-1*, *ScCBL3-1*, and *ScCBL4*, respectively. Basic information about these three genes is shown in Table 1. The length of the *ScCBLs* open reading frames (ORFs) ranged from 642 to 678 base pairs, and they encoded polypeptides from 213 to 225 amino acids in length. The isoelectric points (pIs) of the polypeptides ranged from 4.77 to 4.82, and the grand average of hydropathicity (GRAVY) of each ScCBL was negative. The molecular weight (MW) of these ScCBLs ranged from 24.31 to 25.85 kDa.

**Table 1:** Features of *CBL* genes in sugarcane.

Gene	GenBank Acc No.	ORF Length (bp)	Peptide Length	MW (kDa)	PI	GRAVY
*ScCBL2-1*	KX013374	678	225	25.79	4.82	−0.208
*ScCBL3-1*	KX013375	678	225	25.85	4.8	−0.234
*ScCBL4*	KX013376	642	213	24.31	4.77	−0.275

Notes: MW, molecular weight; PI, isoelectric point; GRAVY, grand average of hydropathicity.

### Phylogenetic analysis of ScCBLs

**Figure 1:**
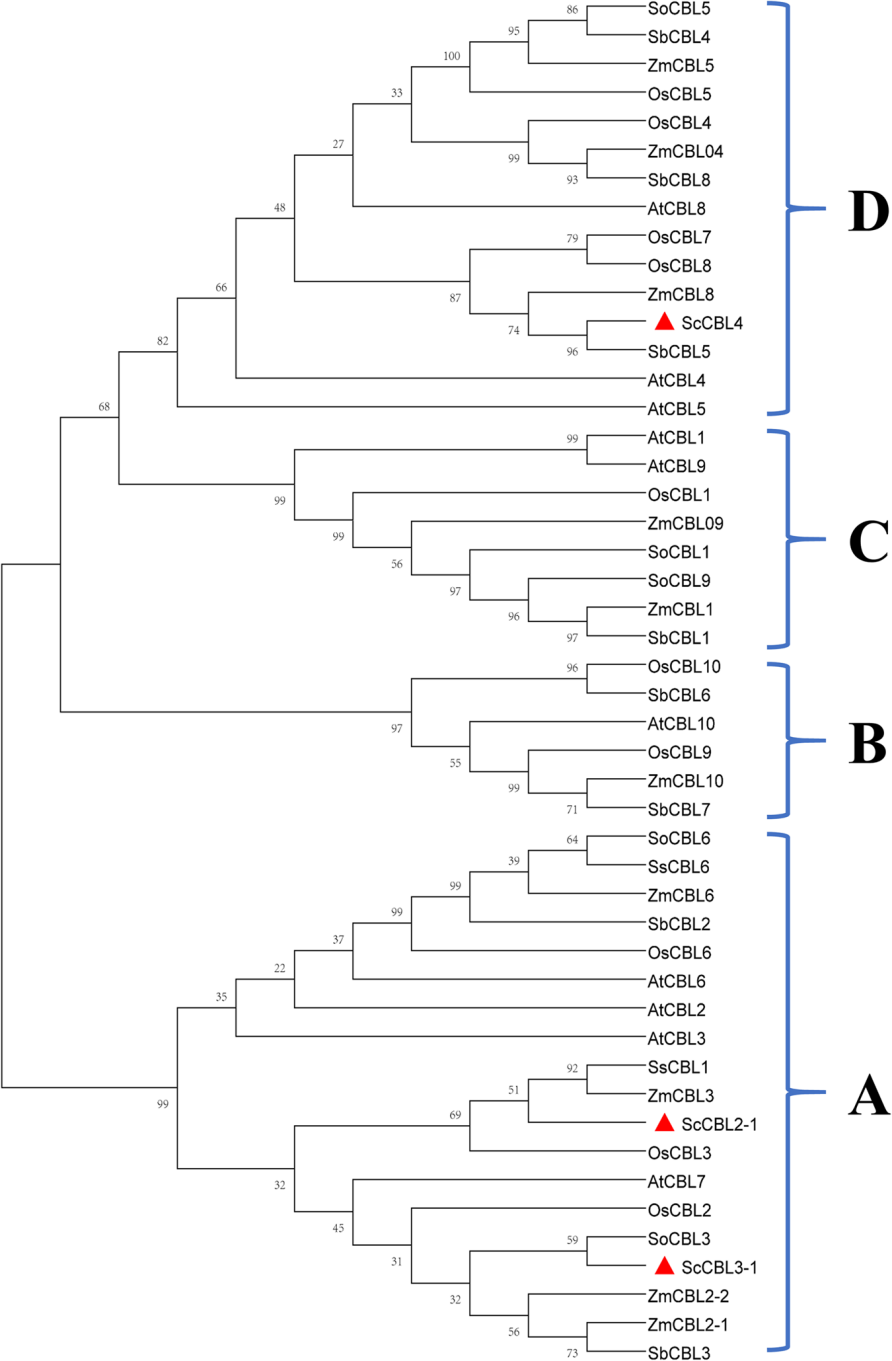
Phylogenetic analysis of the predicted amino acid sequence of CBL proteins from sugarcane and other plant species. The phylogenetic tree was generated using MEGA X software, and the sequence phylogram was constructed by the neighbor-joining bootstrap method (1,000 replicates). At, *Arabidopsis thaliana*; Os, *Oryza sativa*; Zm, *Zea mays*; Sb, *Sorghum bicolor*; So, *Saccharum* spp. (GT28); Ss, *Saccharum* spp. (ROC22); Sc, *Saccharum* spp. (ROC22). ScCBLs in this study were indicated in red triangles. All the corresponding GenBank Accession numbers were listed in Table S3.

Sequences for CBL proteins identified in *A. thaliana*^11,42^, *O. sativa*^11,24^, *Zea mays*^43^, and *S. bicolor*^44^ were obtained from GenBank (https://www.ncbi.nlm.nih.gov/). A phylogenetic tree analysis was performed and the CBL proteins were grouped into four clades (A–D; see Fig. 1). The three sugarcane CBL proteins fell into two different groups: ScCBL2-1 and ScCBL3-1 were in group A, and ScCBL4 was in group D.

### Sequence analysis of the ScCBL proteins

**Figure 2:**
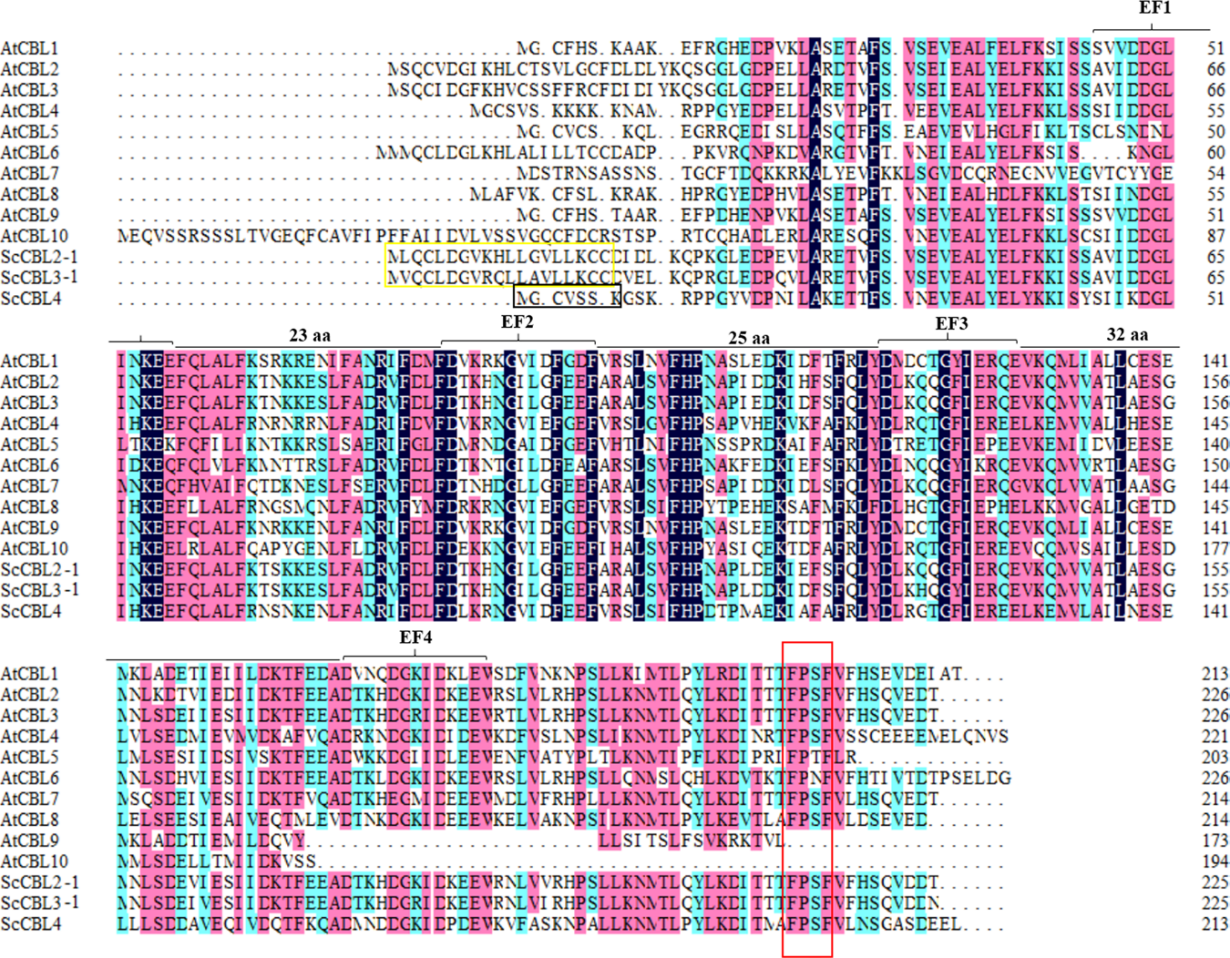
Sequence homology analysis of sugarcane and *A. thaliana* CBLs. The black box indicated the ScCBL4 myristoylation site. The yellow box indicated the ScCBL2-1 and ScCBL3-1 tonoplast targeting sequences. The red box indicated the FPSF motif. The EF notations indicated the EF-hand domain. Sequences highlighted in dark blue, red, and light blue indicated homology 100%, ≥ 75%, and ≥ 50% homology, respectively. aa, amino acids. All the corresponding GenBank Accession were listed in Table S3.

A multiple alignment analysis was performed using the amino acid sequences of the three ScCBLs and ten AtCBLs^11^. As shown in Figure 2, all of these CBL proteins contained more than two EF-hand domains, which are essential for CBL to bind Ca^2+^^19^. The C-terminal region of all three ScCBL proteins contained an FPSF motif. Note that only ScCBL4 contained an N-terminal MGCVSSK sequence, which is a unique CBL protein domain referred to as the myristoylation domain^45,46^. ScCBL2-1 and ScCBL3-1 had N-terminal tonoplast targeting sequences (TTSs), which may mediate their subcellular localization^47^. The size of the linker regions between the EF-hand loops was absolutely invariant in all three of the proteins: 23 amino acids separated EF1 and EF2, whereas 25 amino acids separated EF2 and EF3, and 32 amino acids separated EF3 and EF4.

### Tissue-specific expression of the *ScCBL* genes

**Figure 3:**
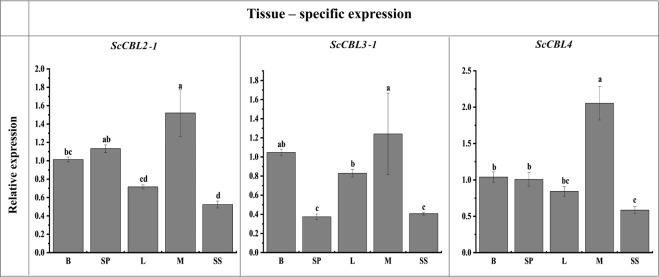
Expression of *ScCBL* genes in different sugarcane tissues, as assessed by qRT-PCR. Data were normalized to cullin (*CUL*) and clathrin adaptor complex (*CAC*) expression levels. All data points were means ± SE (n = 3). Different lowercase letters indicated a significant difference, as determined by the least-significant difference test (*p*-value < 0.05). B, bud; SP, stem pith; L, leaf; M, meristem; SS, stem skin.

*ScCBL* expression in different sugarcane tissues (bud, stem pith, leaf, meristem, and stem skin) was detected by qRT-PCR. Figure 3 shows that the three *ScCBLs* were expressed in all of the tissues tested. *ScCBL2-1*, *ScCBL3-1*, and *ScCBL4* were expressed at the highest levels in the meristem. *ScCBL2-1* and *ScCBL4* were expressed at the lowest levels in the stem skin, while *ScCBL3-1* had the lowest transcription in the stem pith.

### Expression of the *ScCBL* genes in response to phytohormones and various abiotic stresses

**Figure 4:**
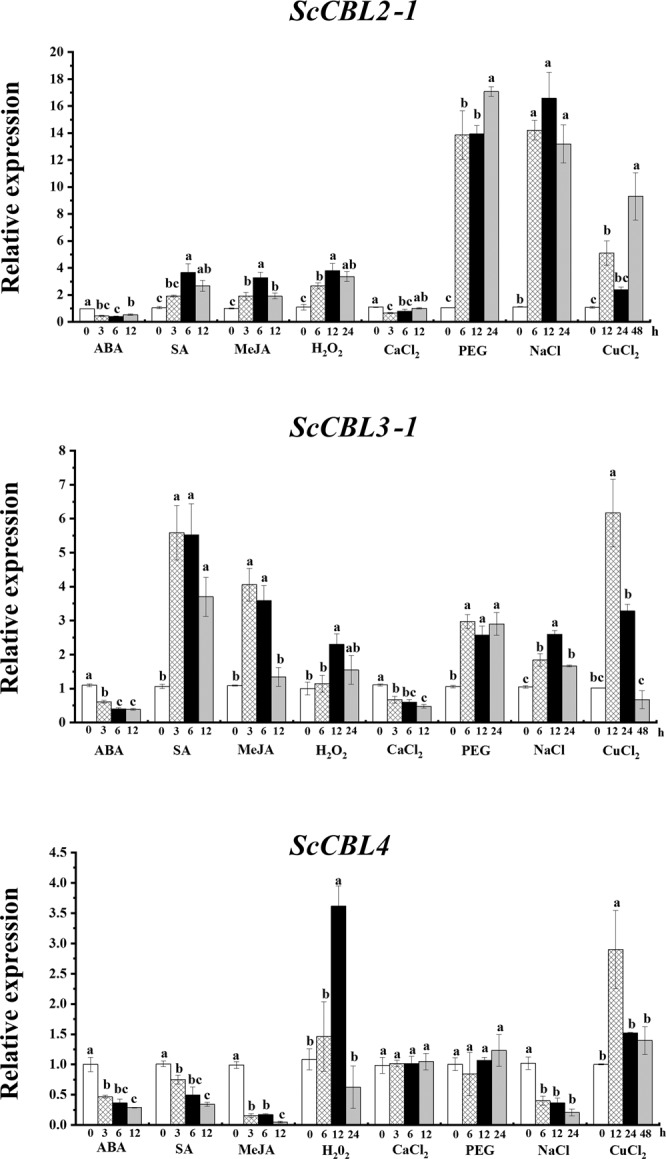
Expression of *ScCBL* genes in ROC22 plantlets after treatment with 100 μM ABA, 5 mM SA, 100 μM MeJA, 10 μM H_2_O_2_, 50 μM CaCl_2_, 25% PEG, 250 mM NaCl, or 100 mM CuCl_2_, as determined by qRT-PCR. Data were normalized to the cullin (*CUL*) and clathrin adaptor complex (*CAC*) expression levels. All data points were means ± SE (n = 3). Different lowercase letters indicate a significant difference, as determined by the least-significant difference test (*p*-value < 0.05). ABA, abscisic acid; SA, salicylic acid; MeJA, methyl jasmonate; H_2_O_2_, hydrogen peroxide; CaCl_2_, calcium chloride; PEG, polyethylene glycol; NaCl, sodium chloride; CuCl_2_, copper chloride.

qRT-PCR analysis showed that the three *ScCBL* genes exhibited different expression patterns in response to ABA, SA, MeJA, H_2_O_2_, CaCl_2_, PEG, NaCl, or CuCl_2_ stress (Fig. 4). When subjected to ABA stress, *ScCBL2-1*, *ScCBL3-1* and *ScCBL4* transcription were all inhibited. Under SA, *ScCBL2-1* and *ScCBL3-1* were expression-induced, but *ScCBL4* was down-regulated. As for MeJA, the three genes have the similar expression pattern with that under SA. Handling with H_2_O_2_, the expression of all *ScCBLs* peaked at 12 h. *ScCBL2-1* and *ScCBL3-1* expression was inhibited in response to treatment with CaCl_2_. We did not find significant difference, however, in the expression of *ScCBL4* between treatment and control. PEG stress did not induce a significant expression level difference of *ScCBL4* in compared with the control, but *ScCBL2-1* and *ScCBL3-1* expression was up-regulated (*ScCBL2-1* peaked at 24 h at a value 17.1 times higher than that of the control, and *ScCBL3-1* peaked at 6 h at a value 3.0 times higher than that of the control). Treatment with NaCl inhibited *ScCBL4* expression but induced *ScCBL2-1* and *ScCBL3-1* expression, with the highest expression levels (16.7 times and 2.5 times higher than that of the control, respectively) occurring at 12 h. In response to CuCl_2_ treatment, *ScCBL2-1*, *ScCBL3-1*, and *ScCBL4* were up-regulated (*ScCBL2-1* expression sharply increased at 48 h to a value 9.3 times higher than that of the control, and *ScCBL3-1*, and *ScCBL4* expression peaked at 12 h at values that were 6.1 and 2.9 times higher, respectively, than that of the control).

### Subcellular localization of the ScCBL proteins

**Figure 5:**
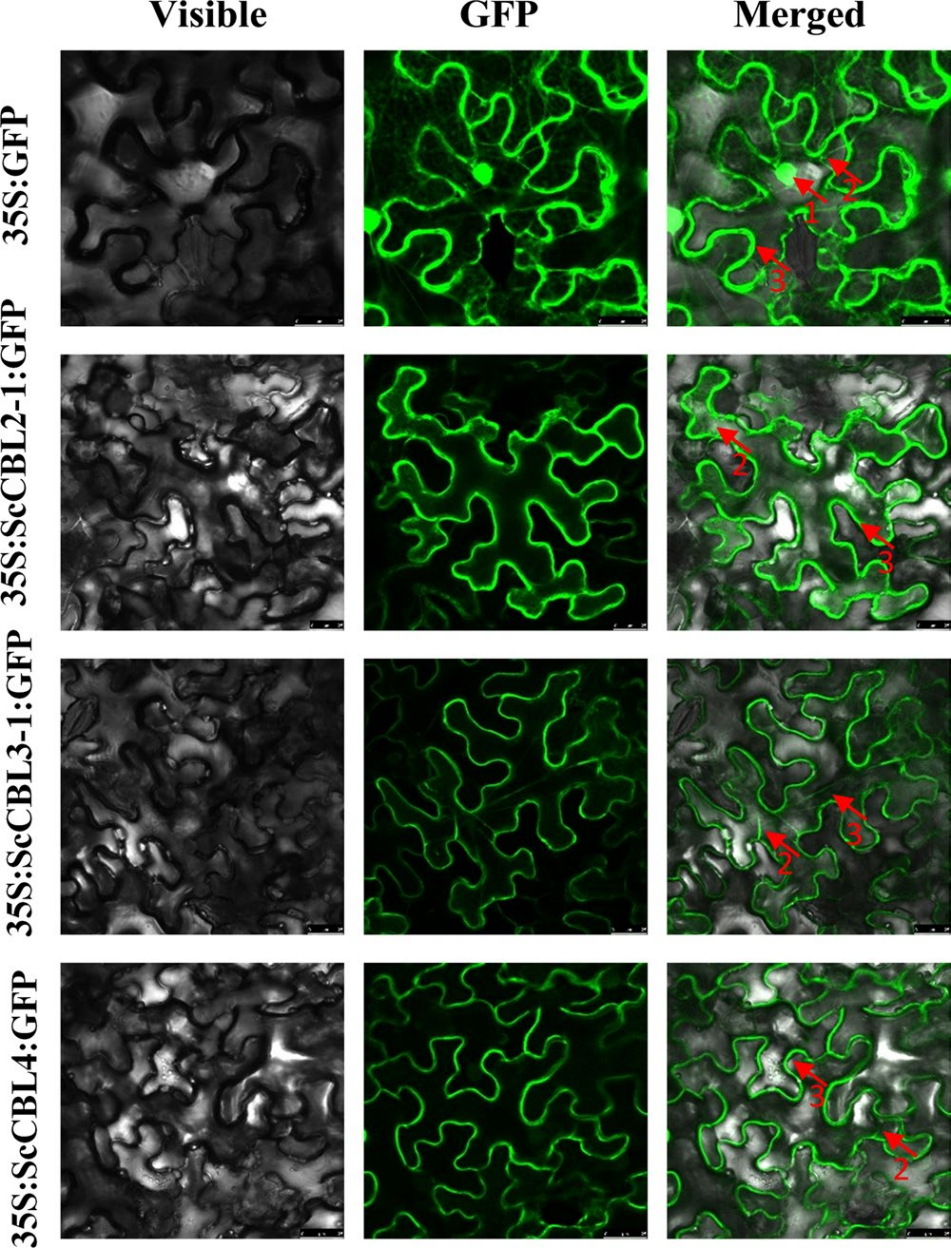
Subcellular localization of ScCBLs in *Nicotiana benthamiana* leaves 48 h after infiltration. Epidermal cells were imaged using visible light, green fluorescence, and merged light. Red arrows labeled 1, 2, and 3 indicated the nucleus, cytoplasm, and plasma membrane, respectively.

To understand the subcellular localization of ScCBLs, ScCBL::GFP fusion proteins were expressed transiently in *N. benthamiana* leaf cells (Fig. 5). At 48 h post-infiltration, ScCBL2-1::GFP, ScCBL3-1::GFP, and ScCBL4::GFP were observed to locate in the plasma membrane and cytoplasm.

### *ScCBL* genes expression in *E. coli* BL21 (DE3) strain

**Figure 6:**
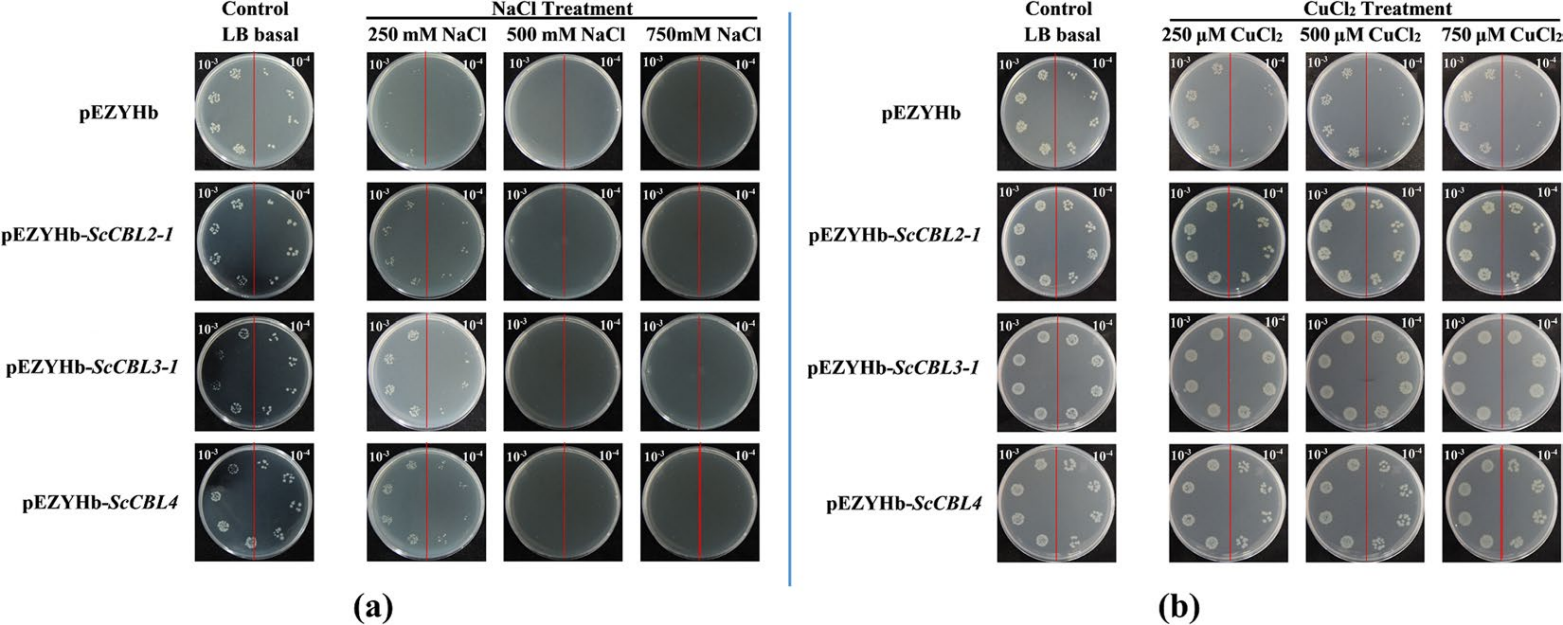
Growth of BL21/pEZYHb and BL21/pEZYHb-*ScCBLs* in *E. coli* BL21 cells. IPTG (1.0 mM) was added to the cultures to induce expression of the recombinant protein. The cultures were diluted 1,000-fold (left side of the red line on the plate) and 10,000-fold (right side of the red line on the plate), and 10 μL of the dilutions were spotted onto LB plates. LB plates without any supplements were used as the control. To explore the impact of BL21/pEZYHb and BL21/pEZYHb-*ScCBLs* on the response to salt and metal stresses, LB plates were supplemented with NaCl (250 mM, 500 mM, and 750 mM) or CuCl_2_ (250 μM, 500 μM, and 750 μM).

Bacterial cells overexpressing pEZYHb-*ScCBLs* had similar growth to control cells on solid LB medium (control), whereas the cells grown on media containing different concentrations of salt or CuCl_2_ showed marked differences in growth (Fig. 6). None of the cells grew on LB plates supplemented with 500 or 750 mM NaCl (Fig. 6(a)). Cells transformed with pEZYHb-*ScCBLs*, however, exhibited better survival on LB plates supplemented with 250 mM NaCl compared with untransformed cells (Fig. 6(a)). These results indicated that bacterial cells overexpressing pEZYHb-*ScCBLs* had better tolerance to high salt stress, which halted the growth of control cells. We monitored the role of pEZYHb-*ScCBLs* in metal stress by supplementing the growth media with various concentrations of CuCl_2_. Cells containing pEZYHb-*ScCBLs* grew more abundantly on LB plates supplemented with CuCl_2_ compared with control cells (Fig. 6(b)), suggesting that bacteria harboring pEZYHb-*ScCBLs* had significant tolerance to CuCl_2_.

### Transient overexpression of *ScCBL* genes in *N. benthamiana* leaves

**Figure 7:**
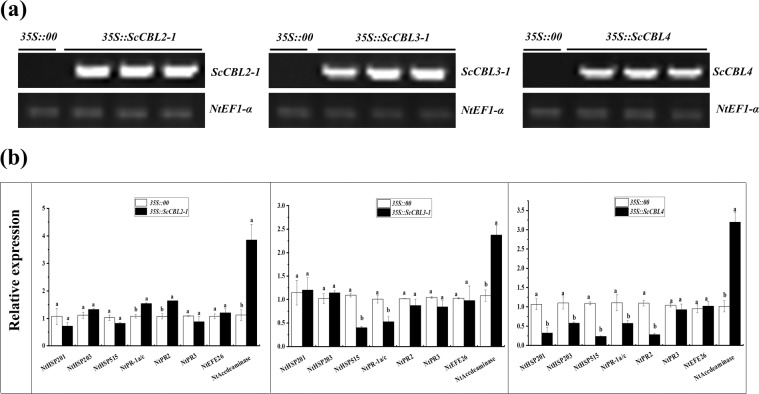
Transient overexpression of *ScCBLs* in *Nicotiana benthamiana* leaves. (**a**) RT-PCR analysis of *ScCBLs* in the *N. benthamiana* leaves at 24 h post-infiltration (The gels were selected from the same gel with the same exposure, and the unmodified figure was shown in Figure S2). (**b**) Relative expression of immunity-associated marker genes in the *N. benthamiana* leaves at 24 h post-infiltration. The transcript levels were normalized to *NtEF1-α*. Mock, the *Agrobacterium* strain carrying *35 S::00*. All data points were means ± SE (n = 3). Different lowercase letters indicated a significant difference, as determined by the Duncan’s new multiple range test (*p*-value < 0.05).

After transient overexpression of *ScCBL* genes in *N. benthamiana* leaves at 24 h post-infiltration, *ScCBL* genes were detected by RT-PCR (Fig. 7(a)). The expression levels of eight immunity-associated marker genes in *N. benthamiana* were detected by qRT-PCR, including the hypersensitivity response (HR) marker genes *NtHSR201*, *NtHSR203*, and *NtHSR515*; the salicylic acid–related gene *NtPR*−*1a/c*; the jasmonic acid pathway–associated genes *NtNPR*2 and *NtNPR3*; and the ethylene synthesis–dependent genes *NtEFE26* and *NtAccdeaminase* (Fig. 7(b)). After 24 h of infiltration, the expression levels of *NtHSR201*, *NtHSR203*, *NtHSR515*, *NtNPR3*, and *NtEFE26* remained unchanged, while *NtPR*-1a/c, *NtNPR2*, and *NtAccdeaminase* were up-regulated in 35* S::ScCBL2-1* leaves. In 35* S::ScCBL3-1* leaves, *NtHSR*515 and *NtPR*-1a/c were down-regulated, but *NtAccdeaminase* was up-regulated. Except for *NtAccdeaminase* was up-regulated, the expression of the other five HR marker genes (*NtHSR201*, *NtHSR203*, *NtHSR515*, *NtPR*-1a/c, and *NtPR2*) was down-regulated in *35 S::ScCBL4* leaves.

**Figure 8:**
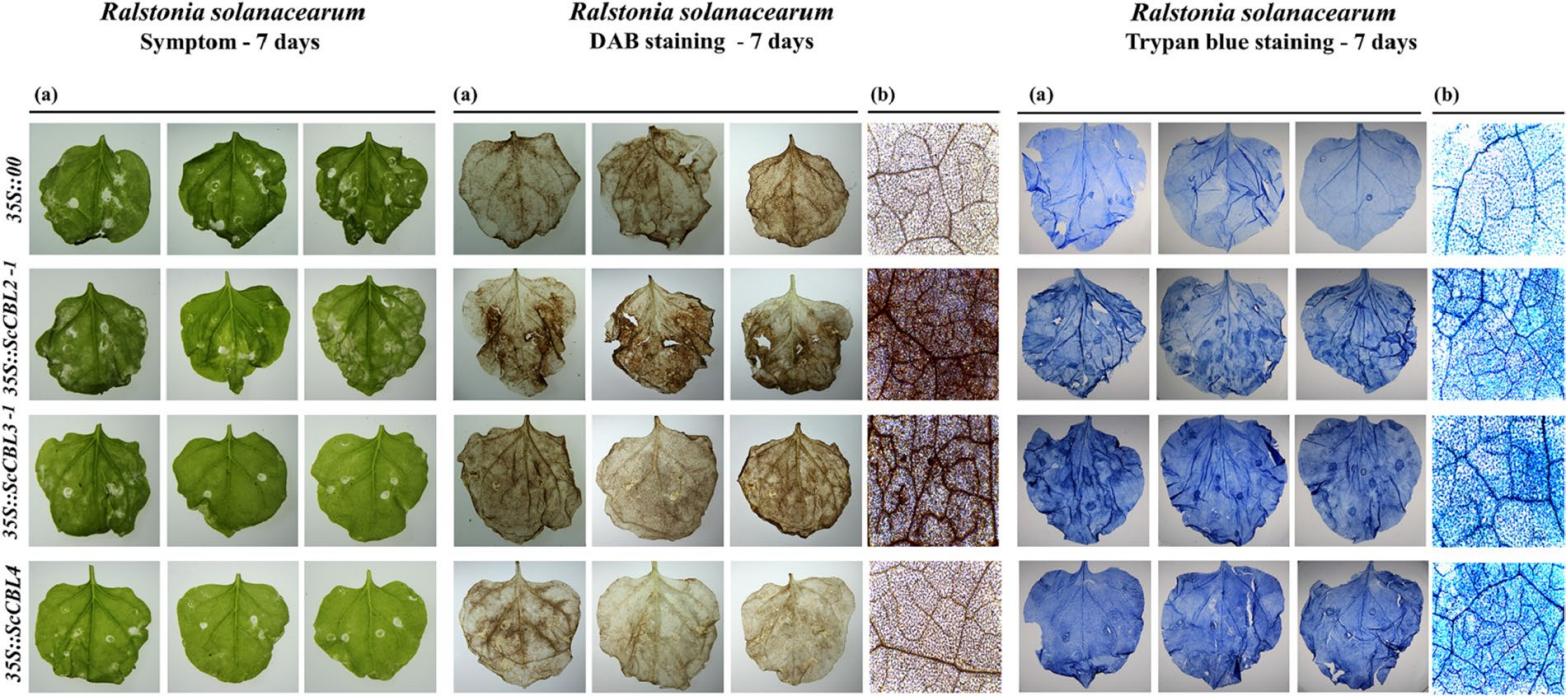
Resistance effect of transient overexpression of *ScCBL* genes in *Nicotiana benthamiana* leaves. Images were captured using a (**a**) stereoscope and (**b**) a light microscope. Disease symptoms, DAB staining and trypan blue staining results from *N. benthamiana* leaves infected with *R. solanacearum* after infiltration with *35* *S::00* (control) or *35* *S::ScCBLs*-containing *Agrobacterium*. Disease symptoms of infected leaves were observed at 7 days post-inoculation.

The results of pathogen infection of tobacco plants transiently expressing the *ScCBLs* are shown in Fig. 8. At 7 days post-inoculation with *R. solanacearum*, we observed disease symptoms in both the *35 S::ScCBLs* and the control (*35 S::00*) leaves, but the yellow necrotic spots on the control leaves were larger than those observed on *35 S::ScCBLs* leaves. In addition, darker 3, 3′-diaminobenzidine (DAB) staining was detected in *35 S::ScCBL2-1* and *35 S::ScCBL3-1* leaves than in the control leaves. And there were more intensely trypan blue–stained cells in *35 S::ScCBLs* leaves than in the control leaves.

## Discussion

Studies show that plant signal transduction processes under stress are accompanied by changes in cellular calcium concentration^48,49^. As a unique Ca^2+^ sensor in plants, CBL plays an important role in signal pathways of plant development and response to various stresses^50^. In this study, we characterized and discussed the possible functions of *ScCBL2-1*, *ScCBL3-1*, and ScCBL4 from sugarcane based on the results of bioinformatic analysis and experiments.

### Sequences and phylogenetic analysis of ScCBLs

In the present study, three *CBL* genes were isolated from sugarcane. The ScCBL proteins appear to be rather conserved in size and structure. These *ScCBL* genes were predicted to encode polypeptides ranging from 24.31 to 25.85 kDa. The results are similar to those in *A. thaliana* and *O. sativa*, in which most AtCBLs and OsCBLs ranged from 23.5 to 25.9 kDa in size^11^. The sequence comparison results showed that the three ScCBL proteins all contained a C-terminal FPSF motif, and the serine residue in these FPSF motifs could be phosphorylated by the CIPK protein kinase^51,52^. A TTS motif, which mediates subcellular localization^47^, was clearly seen in the N-terminus of ScCBL2-1 and ScCBL3-1. The N-terminus of ScCBL4 contained a MGCVSSK sequence, which is a unique CBL protein domain known as the myristoylation domain^45,46^. This domain has been hypothesized to be the ancestral localization domain for CBLs^53^. The TTS motif of ScCBL2-1 and ScCBL3-1 had the same consensus motif as that found in AtCBL2 and AtCBL3, which spanned 19 amino acids^47^. The TTS in AtCBL2 or AtCBL3 was necessary and sufficient for targeting GFP fusion proteins to the tonoplast in *A. thaliana* mesophyll cells^47^. We thus speculate that ScCBL2-1 and ScCBL3-1 may have the same function to AtCBL2 or AtCBL3^47^. However, we found that ScCBL2-1 and ScCBL3-1 not only located in the plasma membrane, but also in the cytoplasm. Phylogenetic analysis placed the three ScCBL proteins into two clades. The ScCBL members investigated here clustered closely with SoCBL, SsCBL, ZmCBL, SbCBL, and OsCBL orthologs, which indicated that the closer evolutionary relationship between the four species from the gramineae family (*Saccharum* spp., *S. bicolor*, *Z. may*, and *O. sativa*) compared to those of *A. thaliana*. In addition, interestingly, we found that CBL family members in *A. thaliana* (AtCBL1, 4, 5, and 9) and *O. sativa* (OsCBL1, 4, 5, 7, and 8) that harbor an N-myristoylation motif were distributed into two neighboring subgroups of the phylogenetic tree (C and D), and ScCBL4 fell into subgroup D. This site diversity may have enabled the evolutionary separation of CBL-type membrane-associated and membrane-independent calcium signaling pathways^11^.

### Differential responses of *ScCBLs* to phytohormones and abiotic stresses

Several studies have shown that *CBL* genes play an important role in the plant stress response^12,21,43,54^. ABA, SA and MeJA, which are phytohormones, play an important role in the response of plants to adverse environmental conditions^55^. H_2_O_2_ is a ROS molecule that mediates signaling functions^56^. Through the interaction with CIPK, CBL protein regulates the production of H_2_O_2_ in the presence of NADPH oxidase, so as to maintain the positive feedback mechanism of stress tolerance^57^. In this study, when subjected to ABA stress, the three *ScCBLs* genes were all down-regulated. In *Arabidopsis*, *AtCBL2* and *AtCBL3* were also not obviously altered by ABA^47^. Under SA and MeJA, *ScCBL2-1* and *ScCBL3-1* were up-regulated while *ScCBL4* was inhibited. *PsCBL* which is orthologues to *AtCBL2*, was up-regulated in response to SA^18,58^. And the expression of *ScCBLs* was induced under H_2_O_2_. We guess that the up-regulated *ScCBLs* were in response to the regulation of exogenous H_2_O_2_. Besides, CaCl_2_ stress did not induce any significant change in *ScCBL4* expression, whereas *ScCBL2-1* and *ScCBL3-1* were down-regulated. Recent biophysical evidence has indicated that Ca^2+^ does not stimulate the interaction between CBL2 and CIPK14, even though Ca^2+^ is required for kinase activation through CBL^59,60^. Different *ScCBL* genes showed various expression patterns in response to CaCl_2_ stress, so the interaction between these *ScCBL* genes and Ca^2+^ needs to be further investigated. We also found that *ScCBL2-1* and *ScCBL3-1* were up-regulated in response to PEG, NaCl, and CuCl_2_, while *ScCBL*4 was induced by CuCl_2_, and inhibited by NaCl. Studies have shown that *ZmCBL4* can significantly improve the salt tolerance of transgenic *Arabidopsis*^43^. *AtCBL2* and *AtCBL3* were marginally induced by dehydration^47^. From all the above, we deduced that *ScCBL* genes have different expression patterns in response to various stresses. We thus speculate that the same or different expression patterns among family genes may be caused by the functional divergence during evolution, which is accordance with the previous research that a homologous pattern resulted from genome duplication, and it caused the gain or loss of function as part of fine-tuning cellular function due to new functionalization in the course of genome evolution^61^.

### Prokaryotic expression of *ScCBLs* under NaCl and CuCl_2_

Previous studies showed that *CBL1*, *CBL4*, *CBL9*, and *CBL10* play important roles in the response to high salt stress^46^, for example, *ZmCBL4* can significantly improve the salt tolerance of transgenic *A. thaliana*^43^*. AtCBL10* is mainly induced by salt^62^. At lower concentration of salt (250 mM NaCl), however, *ScCBL*-transformed bacterial cultures showed better survival compared with the untransformed cells. These results suggested that *ScCBL* genes can enhance cell tolerance to low concentrations of salt. Studies have shown that excessive Cu^2+^ can cause oxidative stress, leading to lipid peroxidation, which destroys cell membrane structure^63^. Ca^2+^ can connect phosphates, phospholipids, and protein carboxyl groups on cell membranes, increase the hydrophobicity of cell membranes, and at the same time, reduce membrane permeability and enhance membrane stability^64^. In this study, *ScCBLs*-overexpressing and control bacterial cells had similar growth on solid LB medium (control). Besides, interestingly, in our studies, under metal stress conditions (CuCl_2_), recombinant *ScCBLs* cells exhibited dramatically better survival compared with nonrecombinant cells. These results suggested that *ScCBL* Ca^2+^ sensors can enhance tolerance to CuCl_2_.

### Transient expression of *ScCBLs* response to *R. solanacearum*

Ethylene is thought to act as an internal signal regulator during plant growth and development, and can respond to external adverse conditions including biotic and abiotic stresses^65^. In addition, Ca^2+^ signaling plays a critical role in the response to biotic and abiotic stimuli^66^. Fagerstedt *et al*. found that an increase in the concentration of Ca^2+^ ions can activate the CBL-CIPK system and cause ethylene-responsive gene activation^67^. In the present study, we found that ethylene synthesis–dependent immunity-associated marker gene (*NtAccdeaminase*) was up-regulated when transiently overexpressed *ScCBLs* in *N. benthamiana* leaves (Fig. 7). Moreover, since CBL proteins can function as Ca^2+^ sensor relays^7^, we can hypothesize that *ScCBL* genes may take part in the ethylene synthesis pathway and play a role in the response to external stressors^67^. Reactive oxygen species (ROS) act as signaling molecules to regulate development and stress responses^68^. As a relatively stable active oxygen, H_2_O_2_, plays different roles in plant responses to external stresses^69^. A previous study showed that, in plants, attempted infection by microbial pathogens is often accompanied by rapid cell death in and around the initial infection site and that this response is associated with restricted pathogen growth and represents a form of PCD^70^. In this study, to investigate changes in *ScCBLs* expression in response to pathogen infection, we injected *R. solanacearum* into *N. benthamiana* containing *35 S::ScCBLs* and a control construct. Then, we used DAB staining and trypan blue staining to detect hydrogen peroxide (H_2_O_2_) accumulation and cell necrosis in the leaves. We observed darker DAB staining compared with the control leaves after overexpression of *ScCBL* in *N. benthamiana* leaves and inoculation with *R. solanacearum* (Fig. 8). Besides, we also observed more intense trypan blue staining of cells in *N. benthamiana* leaves overexpressing *ScCBL* genes after inoculation with *R. solanacearum* compared with control leaves. This result suggested that overexpression of *ScCBL* genes can effectively promote resistance to infections in tobacco plants.

## Conclusion

Three *CBL* genes (*ScCBL2-1*, *ScCBL3-1*, and *ScCBL4*) in sugarcane that encode proteins harboring EF-hand motifs were cloned and identified. These *ScCBL* genes were constitutively expressed in the sugarcane bud, stem pith, leaf, meristem, and stem skin. And they showed different expression patterns in response to stimulation with phytohormones and various abiotic stresses. Overexpression of *ScCBL* genes enhanced *E. coli* BL21 cell growth under conditions of NaCl or CuCl_2_ stress. Additionally, transient overexpression of *ScCBL* genes in *N. benthamiana* leaves resulted in different expression levels of tobacco immunity-associated marker genes, as well as increased resistance to infection with *R. solanacearum*. The findings from this study of *ScCBLs* may serve as a basis for the elucidation of the mechanisms underlying sugarcane immunity.

## Supplementary information

Supplementary information.
