# Supplementary material for: Sugarcane calcineurin B-like (CBL) genes play important but versatile roles in regulation of responses to biotic and abiotic stresses
Source: Sci Rep. 2020 Jan 13;10:167. doi: 10.1038/s41598-019-57058-7 (PMC6957512; doi:10.1038/s41598-019-57058-7)
Supplement: Supplementary file 1 — Supplementary information. [file 41598_2019_57058_MOESM1_ESM.pdf]

Sugarcane calcineurin B-like (*CBL*) genes play important but versatile roles in regulation of responses to biotic and abiotic stresses

Su Wei-hua [1], Huang Long [1], Ling Hui [1], Mao Hua−ying [1], Huang Ning [1], Su Ya-chun [1], Ren Yong-juan [1], Wang Dong-jiao [1], Xu Li-ping [1], Khushi Muhammad [2] & Que You-xiong [1,3,*]

[1] Key Laboratory of Sugarcane Biology and Genetic Breeding, Ministry of Agriculture, Fujian Agriculture and Forestry University, Fuzhou 350002, China
[2] Department of Genetics, Hazara University, Mansehra, Pakistan
[3] Guangxi Collaborative Innovation Center of Sugarcane Industry, Guangxi University, Nanning 530005, China

[*]Corresponding author. E-mail: queyouxiong@126.com

Supplementary tables

Table S1 Primers used in this study

**Primer**	**Forward primer (5’-3’)**	**Reverse primer (5’-3’)**	**Strategy**
ScCBL2-1	ATGTTGCAGTGCCTGGATG	GGTATCATCGACCTGAGAAT	Gene cloning
ScCBL3-1	GCCTGCCCAACTCGATTCT	ACTCGAATCCAGCATGCAAC	Gene cloning
ScCBL4	GTGCTTGCTTTGGTCGCTT	GTTGCATCCTTCAGTGCGTT	Gene cloning
qScCBL2-1	TGAGGAGGCAGACACAAG	CGACCTGAGAATGGAAGACA	qRT-PCR analysis
qScCBL3-1	ATGGTTGTTGCGACACTTGC	AGCAAGGATGGATGCCGAAT	qRT-PCR analysis
qScCBL4	GCAAGCAGACATGAACGACG	GTGTTCCTTCCTTCCCAGCA	qRT-PCR analysis
CAC	ACAACGTCAGGCAAAGCAAA	AGATCAACTCCACCTCTGCG-3	qRT-PCR analysis
CUL	TGCTGAATGTGTTGAGCAGC	TTGTCGCGCTCCAAGTAGTC	qRT-PCR analysis
gScCBL2-1	GGGGACAAGTTTGTACAAAAAAGCAGGCTTC<br>ATGTTGCAGTGCCTGGATGG	GGGGACCACTTTGTACAAGAAAGCTGGGTCG<br>GTATCATCGACCTGAGAAT	Gateway entry vector construction and RT-PCR analysis
gScCBL3-1	GGGGACAAGTTTGTACAAAAAAGCAGGCTTC<br>ATGGTGCAGTGCCTGGACGG	GGGGACCACTTTGTACAAGAAAGCTGGGTCG<br>TTATCATCGACTTGAGAGT	Gateway entry vector construction and RT-PCR analysis
gScCBL4	GGGGACAAGTTTGTACAAAAAAGCAGGCTTC<br>ATGGGGTGTGTCTCCTCCAA	GGGGACCACTTTGTACAAGAAAGCTGGGTCC<br>AACTCTTCATCACTAGCTC	Gateway entry vector construction and RT-PCR analysis
NtHSR201	CAGCAGTCCTTTGGCGTTGTC	GCTCAGTTTAGCCGCAGTTGTG	qRT-PCR analysis
NtHSR203	TGGCTCAACGATTACGCA	GCACGAAACCTGGATGG	qRT-PCR analysis
NtHSR515	TTGGGCAGAATAGATGGGTA	TTTGGTGAAAGTCTTGGCTC	qRT-PCR analysis
NtPR-1a/c	AACCTTTGACCTGGGACGAC	GCACATCCAACACGAACCGA	qRT-PCR analysis
NtPR2	TGATGCCCTTTTGGATTCTATG	AGTTCCTGCCCCGCTTT	qRT-PCR analysis
NtPR3	CAGGAGGGTATTGCTTTGTTAGG	CGTGGGAAGATGGCTTGTTGTC	qRT-PCR analysis
NtEFE26	CGGACGCTGGTGGCATAAT	CAACAAGAGCTGGTGCTGGATA	qRT-PCR analysis
NtAccdeaminase	TCTGAGGTTACTGATTTGGATTGG	TGGACATGGTGGATAGTTGCT	qRT-PCR analysis
NtEF1-α	TGCTGCTGTAACAAGATGGATGC	GAGATGGGGACAAAGGGGATT	qRT-PCR analysis

Note: attB1 and attB2 adapters were underlined in the primer *gScCBLs*.

Table S2 The amplification reaction procedures for cloning of sugarcane *ScCBLs*

Gene name	Pre-denaturation	Denaturation	Annealing	Extension	Cycles	Extension
ScCBL2-1	94 °C 4 min	94 °C 1 min	55 °C 30 s	72 °C 80 s	35	72 °C 10 min
ScCBL3-1	94 °C 4 min	94 °C 30 s	55 °C 30 s	72 °C 2 min	35	72 °C 10 min
ScCBL4	94 °C 4 min	94 °C 30 s	55 °C 30 s	72 °C 2 min	35	72 °C 10 min

Table S3 GenBank Accession Numbers used in this study

Species name	Gene name	GenBank Accession Numbers	Species name	Gene name	GenBank Accession Numbers
Arabidopsis thaliana	AtCBL1	AAC26008	*Oryza sativa*	OsCBL1	DQ201195
	AtCBL2	AAC26009		OsCBL2	DQ201196
	AtCBL3	AAC26010		OsCBL3	DQ201197
	AtCBL4	AAC26110		OsCBL4	DQ201198
	AtCBL5	CAB80951		OsCBL5	DQ201199
	AtCBL6	AAG28400		OsCBL6	DQ201200
	AtCBL7	AAG10059		OsCBL6	DQ201201
	AtCBL8	AAL10300		OsCBL8	DQ201202
	AtCBL9	AAL10301		OsCBL9	DQ201203
	AtCBL10	AAO72364		OsCBL10	DQ201204
Zea mays	ZmCBL1	EU907931	*Sorghum bicolor*	SbCBL1	FJ901259
	ZmCBL2-1	EU907932		SbCBL2	FJ901264
	ZmCBL2-2	EU907933		SbCBL3	FJ901265
	ZmCBL3	EU907934		SbCBL4	FJ901261
	ZmCBL5	EU907935		SbCBL5	FJ901263
	ZmCBL6	EU907936		SbCBL6	FJ901262
	ZmCBL8	EU907937		SbCBL7	FJ901260
	ZmCBL10	EU907938		SbCBL8	FJ901266
Saccharum spp. (ROC22)	SsCBL1	KC800815.1	*Saccharum* spp. (GT28)	SoCBL1	KC800815
	ScCBL2-1	KX013374		SoCBL3	KC800816
	ScCBL3-1	KX013375		SoCBL5	KC800817
	ScCBL4	KX013376		SoCBL6	KC800818
	SsCBL6	KC800818.1		SoCBL9	KC800819

Supplementary figures

```
AtCBL2      .......................MSQCVDGIKHLCTSVLGCFDLDLYK.QSGGLGDPELLARDTVFS.VSEIEALYELFK     55
AtCBL3      .......................MSQCIDGFKHVCSSFFRCFDIDIYK.QSGGLGDPELLARETVFS.VSEIEALYELFK     55
ScCBL2-1    .......................MLQCLDGVKHLLGVLLKCCDIDLK..QPKGLEDPEVLARETVFS.VSEVEALYELFK     54
ScCBL3-1    .......................MVQCLDGVRQLLAVLLKCCDVELK..QPRGLEDPQVLARETVFS.VSEVEALYELFK     54
AtCBL1      ..................................MG.CFHSKAAK....EFRGHEDPVKLASETAFS.VSEVEALFELFK     40
AtCBL9      ..................................MG.CFHSTAAR....EFPDHENPVKLASETAFS.VSEVEALYELFK     40
AtCBL10     MEQVSSRSSSLTVGEQFCAVFIPFFAIIDVLVSSVGQCFDCRSTSP...RTCQHADLERLARESQFS.VNEVEALYELFK     76
AtCBL4      ..................................MG.CSVSKKKKKNAMRPPGYEDPELLASVTPFT.VEEVEALYELFK     44
ScCBL4      ..................................MG.CVSSKGSK....RPPGYVDPNILAKETTFS.VNEVEALYELYK     40
AtCBL8      ..............................MLAFVK.CFSLKRAK....HPRGYEDPHVLASETPFT.VNEIEALHDLFK     44
AtCBL5      ..................................MG.CVCSKQLE.....GRRQEDISLLASQTFFS.EAEVEVLHGLFI     39
AtCBL6      ......................MMMQCLDGLKHLALILLTCCDAD....PPKVRQNPKDVARGTVFT.VNEIEALYELFK     53
AtCBL7      ..................................MDSTRNSASSNS...TGCFTDQKKRKALYEVFKKLSGVDCQRNEGN     43
Consensus                                                                a     f

AtCBL2      KISSAVIDDGLINKEEFQLALFKTNKKESLFADRVFDLFDTKHNGILGFEEFARALSVFHPNAPIDDKIHFSFQLYDLKQ    135
AtCBL3      KISSAVIDDGLINKEEFQLALFKTNKKESLFADRVFDLFDTKHNGILGFEEFARALSVFHPNAPIEDKIDFSFQLYDLKQ    135
ScCBL2-1    KISSAVIDDGLINKEEFQLALFKTSKKESLFADRVFDLFDTKHNGILGFEEFARALSVFHPNAPLDEKIEFSFQLYDLKQ    134
ScCBL3-1    KISSAVIDDGLINKEEFQLALFKTSKKESLFADRVFDLFDTKHNGILGFEEFARALSVFHPNAPLDDKIDFSFQLYDLKH    134
AtCBL1      SISSSVVDDGLINKEEFQLALFKSRKRENIFANRIFDMFDVKRKGVIDFGDFVRSLNVFHPNASLEDKIDFTFRLYDMDC    120
AtCBL9      SISSSVVDDGLINKEEFQLALFKNRKKENLFANRIFDLFDVKRKGVIDFGDFVRSLNVFHPNASLEEKTDFTFRLYDMDC    120
AtCBL10     KLSCSIIDDGLIHKEELRLALFQAPYGENLFLDRVFDLFDEKKNGVIEFEEFIHALSVFHPYASIQEKTDFAFRLYDLRQ    156
AtCBL4      KLSSSIIDDGLIHKEEFQLALFRNRNRRNLFADRIFDVFDVKRNGVIEFGEFVRSLGVFHPSAPVHEKVKFAFKLYDLRQ    124
ScCBL4      KISYSIIKDGLIHKEEFQLALFRNSNKENLFANRIFDLFDLKRNGVIDFEEFVRSLSIFHPDTPMAEKIAFAFRLYDLRG    120
AtCBL8      KLSTSIINDGLIHKEEFLLALFRNGSMQNLFADRVFYMFDRKRNGVIEFGEFVRSLSIFHPYTPEHEKSAFMFKLFDLHG    124
AtCBL5      KLTSCLSNDNLLTKEKFQFILIKNTKKRSLSAERIFGLFDMRNDGAIDFGEFVHTLNIFHPNSSPRDKAIFAFRLYDTRE    119
AtCBL6      SIS....KNGLIDKEQFQLVLFKMNTTRSLFADRVFDLFDTKNTGILDFEAFARSLSVFHPNAKFEDKIEFSFKLYDLNQ    129
AtCBL7      VVEGVTCYYGEMNKEQFHVAIFQTDKNESLFSERVFDLFDTNHDGLLGFEEFARALSVFHPSAPIDDKIDLSFQLYDLKQ    123
Consensus                ke                r f  fd    g    f  f    l   fhp      k    f l d

AtCBL2      QGFIERQEVKQMVVATLAESGMNLKDTVIEDIIDKTFEEADTKHDGKIDKEEWRSLVLRHPSLLKNMTLQYLKDITTTFP    215
AtCBL3      QGFIERQEVKQMVVATLAESGMNLSDEIIESIIDKTFEEADTKHDGRIDKEEWRTLVLRHPSLLKNMTLQYLKDITTTFP    215
ScCBL2-1    QGFIERQEVKQMVVATLAESGMNLSDEVIESIIDKTFEEADTKHDGKIDKEEWRNLVVRHPSLLKNMTLQYLKDITTTFP    214
ScCBL3-1    QGYIERQEVKQMVVATLAESGMNLSDEIVESIIDKTFEEADTKHDGRIDKEEWRNLVIRHPSLLKNMTLQYLKDITTTFP    214
AtCBL1      TGYIERQEVKQMLIALLCESEMKLADETIEIILDKTFEDADVNQDGKIDKLEWSDFVNKNPSLLKIMTLPYLRDITTTFP    200
AtCBL9      TGFIERQEVKQMLIALLCESEMKLADDTIEMILDQVYLLSIT...........SLFSVKRKTVL................    173
AtCBL10     TGFIEREEVQQMVSAILLESDMMLSDELLTMIIDKVSS..........................................    194
AtCBL4      TGFIEREELKEMVVALLHESELVLSEDMIEVMVDKAFVQADRKNDGKIDIDEWKDFVSLNPSLIKNMTLPYLKDINRTFP    204
ScCBL4      TGFIEREELKEMVLAILNESELLLSDDAVEQIVDQTFKQADMNDDGKIDPDEWKVFASKNPALLKNMTLPYLKDITMAFP    200
AtCBL8      TGFIEPHELKKMVGALLGETDLELSEESIEAIVEQTMLEVDTNKDGKIDEEEWKELVAKNPSILKNMTLPYLKEVTLAFP    204
AtCBL5      TGFIEPEEVKEMIIDVLEESELMLSESIIDSIVSKTFEEADWKKDGIIDLEEWENFVATYPLTLKNMTIPFLKDIPRIFP    199
AtCBL6      QGYIKRQEVKQMVVRTLAESGMNLSDHVIESIIDKTFEEADTKLDGKIDKEEWRSLVLRHPSLLQNMSLQHLKDVTKTFP    209
AtCBL7      QGFIERQGVKQLVVATLAASGMSQSDEIVESIIDKTFVQADTKHEGMIDEEEWMDLVFRHPLLLKNMTLQYLKDITTTFP    203
Consensus    g i            l

AtCBL2      SFVFHSQVEDT......                                                                   226
AtCBL3      SFVFHSQVEDT......                                                                   226
ScCBL2-1    SFVFHSQVDDT......                                                                   225
ScCBL3-1    SFVFHSQVDDN......                                                                   225
AtCBL1      SFVFHSEVDEIAT....                                                                   213
AtCBL9      .................                                                                   173
AtCBL10     .................                                                                   194
AtCBL4      SFVSSCEEEEMELQNVS                                                                   221
ScCBL4      SFVLNSGASDEEL....                                                                   213
AtCBL8      SFVLDSEVED.......                                                                   214
AtCBL5      TFLR.............                                                                   203
AtCBL6      NFVFHTIVTDTPSELDG                                                                   226
AtCBL7      SFVLHSQVEDT......                                                                   214
Consensus
```

Figure S1 Sequence homology analysis of sugarcane and *A. thaliana* CBLs. Sequences highlighted in dark blue, red, and light blue indicate homology 100%, ≥75%, and ≥50% homology, respectively. GenBank accession numbers were as follows: AtCBL1 (AAC26008); AtCBL2 (AAC26009); AtCBL3 (AAC26010); AtCBL4 (AAC26110); AtCBL5 (CAB80951); AtCBL6 (AAG28400); AtCBL7 (AAG10059); AtCBL8 (AAL10300); AtCBL9 (AAL10301); AtCBL10 (AAO72364); ScCBL2-1 (KX013374); ScCBL3-1 (KX013375); ScCBL4 (KX013376).

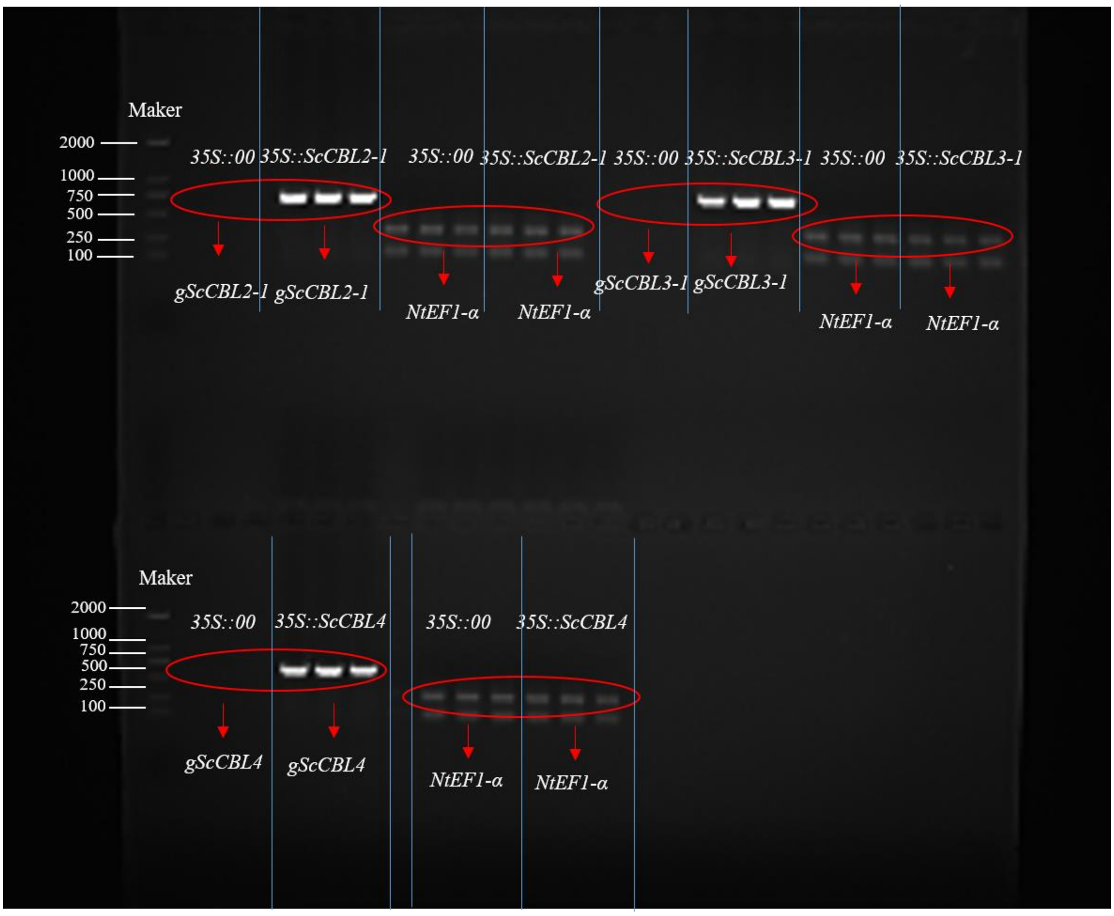

Figure S2 RT-PCR analysis of *ScCBLs* in the *N. benthamiana* leaves at 24 h post-infiltration. Note: *35S::00* stands for the control templet, *35S::ScCBL2-1*, *35S::ScCBL3-1*, and *35S::ScCBL4* stand for the template of instantaneous expression *ScCBL2-1*, *ScCBL3-1*, and *ScCBL4*, respectively. *gScCBL2-1*, *gScCBL3-1*, *gScCBL4* and *NtEF1-α* represented the primer used in the RT-PCR.
